# The Genetic, Environmental, and Immunopathological Complexity of Autoantibody-Negative Rheumatoid Arthritis

**DOI:** 10.3390/ijms222212386

**Published:** 2021-11-17

**Authors:** Ludovico De Stefano, Bernardo D’Onofrio, Antonio Manzo, Carlomaurizio Montecucco, Serena Bugatti

**Affiliations:** 1 Division of Rheumatology, IRCCS Policlinico San Matteo Foundation, Viale Golgi 19, 27100 Pavia, Italy; ludovico.destefano01@universitadipavia.it (L.D.S.); bernardo.donofrio01@universitadipavia.it (B.D.); antonio.manzo@unipv.it (A.M.); montecucco@smatteo.pv.it (C.M.); 2 Department of Internal Medicine and Therapeutics, University of Pavia, Via Aselli 43/45, 27100 Pavia, Italy

**Keywords:** rheumatoid arthritis, seronegative, anti-citrullinated protein antibodies, rheumatoid factor, pathogenesis

## Abstract

Differences in clinical presentation, response to treatment, and long-term outcomes between autoantibody-positive and -negative rheumatoid arthritis (RA) highlight the need for a better comprehension of the immunopathogenic events underlying the two disease subtypes. Whilst the drivers and perpetuators of autoimmunity in autoantibody-positive RA have started to be disclosed, autoantibody-negative RA remains puzzling, also due its wide phenotypic heterogeneity and its possible misdiagnosis. Genetic susceptibility appears to mostly rely on class I HLA genes and a number of yet unidentified non-HLA loci. On the background of such variable genetic predisposition, multiple exogeneous, endogenous, and stochastic factors, some of which are not shared with autoantibody-positive RA, contribute to the onset of the inflammatory cascade. In a proportion of the patients, the immunopathology of synovitis, at least in the initial stages, appears largely myeloid driven, with abundant production of proinflammatory cytokines and only minor involvement of cells of the adaptive immune system. Better understanding of the complexity of autoantibody-negative RA is still needed in order to open new avenues for targeted intervention and improve clinical outcomes.

## 1. Introduction

Rheumatoid arthritis (RA) is a multifactorial chronic immune-inflammatory disease characterized by significant heterogeneity in clinical presentation and outcomes among different individuals with the same formal diagnosis. A major subclassification is based on the presence or absence of classical RA-associated autoantibodies, such as rheumatoid factor (RF) and anti-citrullinated protein antibodies (ACPAs). Traditionally, autoantibody-positive RA, if left untreated, is associated with worst outcomes in terms of high disease activity, rapidly progressive joint damage, and increased mortality [1,2]. In the past 10 years, however, earlier diagnosis and more intensive management with conventional synthetic (cs), biologic (b), and targeted synthetic (ts) disease-modifying anti-rheumatic drugs (DMARDs) have considerably improved the prognosis of RA, with milder disease course achieved in particular in autoantibody-positive patients [3]. In contrast, patients with autoantibody-negative RA still experience delayed diagnosis [4] and highly heterogeneous response to therapy [5], and some continue to develop extensive joint destruction and disability. Indeed, over the years, it has become increasingly apparent that autoantibody-negative RA is a more heterogeneous entity than autoantibody-positive RA, and that the current classification criteria and treatment approaches may still be insufficient at conveying favorable outcomes in a proportion of the patients.

The pathophysiological basis underlying the clinical diversity of RA is only partially understood. In particular, it is at present poorly defined whether autoantibody-positive and -negative RA are sustained by heterogeneous immune mechanisms operating variably, albeit with some overlap, in individual patients. Intensive research into the etiopathogenesis of ACPA-positive RA over the last years has substantially clarified the complex interplay between genetic risk factors and environmental susceptibility resulting in dysregulated adaptive immunity with the generation of pathogenic autoantibodies [6,7]. In contrast, the genetic architecture and the predisposing factors of autoantibody-negative RA remain an area of uncertainty, and the relative contribution of innate over adaptive immune pathways has been hypothesized but not formally proven [8,9]. Although RA is currently managed the same way irrespective of the autoantibody profile, a better understanding of the pathophysiological heterogeneity of the disease would definitively improve personalized approaches more focused on specific risk factors and immune pathways. Here, we will revise current advancements in the understanding of the genetic, environmental, and immunopatogenetic complexity of autoantobody-negative RA.

## 2. Genetic Susceptibility

The contribution of genetic factors to the susceptibility of ACPA-positive and ACPA-negative RA was initially estimated to be equivalent in two small twin studies [10,11]. However, in a recent study using large population-representative samples, the heritability calculation was revised and reported to be 50% for ACPA-positive and 20% for ACPA-negative RA [12]. Despite the lowest susceptibility, autoantibody-negative RA has still been reported to significantly co-aggregate with both autoantibody-positive RA and with spondyloarthropathies in first-degree relatives [13]. This finding highlights the complexity of autoantibody-negative RA, with some cases genetically similar to autoantibody-positive RA but possibly with undetected antibodies, and some others etiologically more related to classical seronegative arthritides.

HLA genes typically play the largest contribution to inherited disease susceptibility [14]. The strongest genetic loci reported for autoantibody-positive RA, specifically the shared epitope-containing HLA-DRB1 alleles, are minimally or not associated with autoantibody-negative RA [15]. Rather, HLA associations appear mostly located within the class I region [14] (Table 1). In particular, studies have consistently reported polymorphic aspartate at position 9 of the class I HLA-B protein, which is strongly correlated with the expression of HLA-B*08 [16,17,18,19]. The same studies also identified serine at position 11 of the class II HLA-DRB1 protein. However, this association is partially explained by the existence of a common extended haplotype, the ancestral haplotype 8.1, which contains HLA-B*08 with aspartate at position 9 and DRB1*03 with serine at position 11. The existence of such linkage disequilibrium might justify previously reported DR3 associations with autoantibody-negative RA [20,21]. From a pathophysiological perspective, variations in HLA class I alleles would underscore the possible predominant role of CD8+ over CD4+ T cells in autoantibody-negative RA [22,23]. Still, HLA-DRB1 haplotypes would appear to play a role also in autoantibody-negative RA by shaping the autoantibody response. The presence of basic aminoacids, such as histidine, at position 13 indeed strongly confers risk of ACPA production [24], whilst not charged or negatively charged aminoacids have been reported to be associated with autoantibody-negative juvenile and adult polyarthritis [25]. Genetic polymorphisms within the HLA region, however, explain only a modest proportion of autoantibody-negative RA heritability. Genome-wide association studies have identified a number of non-HLA determinants at confirmed levels of statistical significance. However, most of the genetic associations have been reported in single studies and have not been independently replicated. Overall, it would appear that a number of non-HLA susceptibility loci are more specific of ACPA-positive RA (AFF3, CCR6, CCL21, IL2RA, and CD28), some are shared irrespective of the autoantibody serotype (TNFAIP3, C5orf30, STAT4, ANKRD55, BLK, and PTPN22), and others are more associated with autoantibody-negative RA (CLYBL, SMIM2, SPP1, CLEC16A, IRF5, and DCIR) [16,26,27,28,29]. Of the markers reported in ACPA-negative RA, ANKRD55 is the only locus to be associated at genome-wide significance levels [16,29]. Although the biologic function of ANKRD55 gene products remains unclarified, single nucleotide polymorphisms in ANKRD55 are risk factors for multiple immune diseases, including multiple sclerosis, Crohn’s Disease, diabetes, and inflammatory myopathies [30]. Recently, novel non-additive loci related to the metabolism of vitamin D, such as DHCR7 and IFR4 mutations, have been proposed [31], but their association with autoantibody-negative RA remains to be replicated. It is also worth mentioning that some forms of autoantibody-negative polyarthritis classified as RA may in fact share genetic similarities with autoinflammatory diseases, including mutations in NLRP3, MEFV, NOD2, or TNFAIP3 genes [8].

## 3. Environmental and Lifestyle Factors

Environmental susceptibility factors in autoantibody-negative RA are thought to be diverse and variably present among patients. Numerous pieces of evidence support the precipitating role of environmental and internal factors, such as respiratory diseases, exposure to toxins, dysbiosis, and biological responses to stress, hormones, and drugs, on the development of autoantibody-negative RA in genetically susceptible patients (Table 1). However, established non-genetic risk factors only provide an explanation for risk of RA in a minor part, suggesting that many (familial) risk factors remain to be identified.

### 3.1. Smoking, Other Pulmonary Irritants, and Respiratory Diseases

Numerous studies have established a clear relationship between smoking and the occurrence of autoantibody-positive RA [32,33]. The risk is significantly higher among RF-positive cases irrespective of the genetic background, whilst the association between smoking and ACPA appears most strongly related to HLA-DRB1 alleles [34,35,36]. Accordingly, it has been reported that smoking increases RA susceptibility in individuals carrying the HLA-DRB1 shared epitope regardless of the autoantibody status [37]. However, outside of genetic studies, the association between smoking and autoantibody-negative RA appears either absent [32] or low [33]. Indirectly supporting the non-causative role of smoking on autoantibody-negative RA, recent studies have indicated a temporal trend for increased incidence of RF-negative RA along with reduced smoking habits [38].

In contrast to cigarette smoking, other pulmonary irritants like air pollution [39,40], organic dusts [41], asbestos [42,43], and silica [44] have been observed to be associated with increased risk not only of autoantibody-positive but also of autoantibody-negative RA, with only silica showing a strong association with ACPA-positive RA alone. In keeping with the aforementioned studies on the different gene–environment interactions in RF-positive and ACPA-positive patients [34,35,36], these results would suggest that pulmonary inflammation could favor RA development not only through the formation of neo-antigens, but also via a number of other mechanisms, such as cytokine production, T cell polarization, epigenetic changes, modulation of the local microbiome, and others [45].

A recent epidemiological study in the Swedish Epidemiological Investigation of Rheumatoid Arthritis (EIRA) cohort helps clarifying the specific relationships between pulmonary inflammation, respiratory irritants such as smoking, and serostatus [46]. Pre-existing respiratory diseases were associated with both autoantibody-positive and -negative incident RA, with only small differences between the two. Acute lower airway exposure indeed interacted with smoking only in ACPA-positive subjects. In contrast, upper and lower chronic respiratory diseases, such as rhinitis, asthma, chronic obstructive pulmonary disease, and interstitial lung disease, were associated with risk of RA irrespective of RF and ACPA status, and more strongly in non-smokers. Based on these findings, it would appear that pulmonary inflammation is associated with RA through different, non mutually exclusive, and possibly synergic pathogenetic mechanisms. On the one side, smoking would predominantly drive autoantibody production, amplified in the context of a predisposing genetic background. Other pulmonary irritants and respiratory diseases would instead promote more generalized immune activation favoring the development of both autoantibody-positive and -negative RA.

### 3.2. Microbial Dysbiosis and Mucosal Inflammation

Accumulating evidence from epidemiological and translational studies indicates that alterations in the composition of the microbial flora at mucosal sites may contribute to the development and perpetuation of many immune-mediated inflammatory diseases [47]. The presence of dysbiosis has been demonstrated in the oral cavity, lungs, and gut of patients with RA, and is thought to have effects on inflammation and activation of autoreactive T and B cells at multiple time-points [48]. Of particular relevance to disease pathogenesis, specific bacteria have been associated with citrullinated protein production [49], and perturbations of the mucosal microbiome are already seen in ACPA-positive individuals at risk of developing RA [50,51]. At present, no specific studies have addressed the possible impact of dysbiosis in autoantibody-negative RA. However, interactions of the microbiota with the human immune system occur at multiple levels beyond autoreactive B cell activation, with effects on toll-like receptor (TLR) signaling, neutrophil extracellular traps formation, expansion of mucosa-derived innate immune cells, and polarization of T cells [47,52]. Supporting a role of microbial dysbiosis also in autoantibody-negative RA, mice deficient in interleukin (IL)-1 receptor antagonist show reduced intestinal microbial diversity and develop spontaneous arthritis through TLR activation and T helper 17 induction irrespective of autoantibodies [53]. Furthermore, respiratory diseases associated with chronic bacterial infection have been linked to RA development irrespective of RF and ACPA [46], and treatment-induced modifications of the circulating microbiome have recently been reported to differ between autoantibody-positive and -negative patients [54].

### 3.3. Body Weight and Diet

An association between excess body weight measured by body mass index (BMI) and RA has been suggested in many observational studies with conflicting results, especially in subgroups of different sex, age, or serological status [55,56,57,58,59]. In a small case-control study of 515 RA cases, Pedersen M [55] first reported that the association of being overweight or obese was confined to ACPA-negative RA. This observation was later confirmed by other independent large cohort studies. In the European Prospective Investigation of Cancer, Norfolk (EPIC-Norfolk) study, a BMI ≥30 was associated with a nearly threefold increase in the risk of developing autoantibody-negative inflammatory polyarthritis, with no significant effects on autoantibody-positive cases (HR 1.05, 95%CI 0.61 to 1.79, age and gender adjusted) [56]. A statistically significant association between obesity and risk of ACPA-negative RA was also found in the EIRA cohort but only among women (OR 1.6, 95%CI 1.0–3.3) [57]. These data are in line with a dose–response meta-analysis, in which an increase in RA risk was observed in overweight and obese women (RR 1.11, 95%CI 1.00–1.23, and RR 1.26, 95%CI 1.12–1.40, respectively) and in obese autoantibody-negative subjects (RR 1.47, 95%CI 1.11–1.96) [58]. In these sub-populations, the likelihood of developing RA increased linearly with the increase of BMI. However, differences related to the serological status were not confirmed in two large prospective cohorts, the Nurses’ Health Study (NHS) and Nurses’ Health Study II (NHSII), where the association between being overweight and obese and RA was borderline significant in both autoantibody-negative and -positive subjects (HR 1.37, 95%CI 0.95–1.98, and HR 1.37, 95%CI 0.91–2.09) [59]. Interestingly, risk was stronger and statistically significant among women diagnosed at age 55 years or younger. Although BMI is a poor measure of adiposity, studies on body composition assessed through whole-body dual energy X-ray absorptiometry have shown lower lean mass and higher fat mass in RA patients both in early and established disease [60,61]. Overall, the association between excess body weight and RA is not surprising, given the proinflammatory role of metabolic factors produced by adipose tissue and adipocytes [62]. The selective risk reported for autoantibody-negative RA should instead be interpreted with caution, as pain and inflammatory markers may be increased in obese subjects irrespective of swollen joints [63], with possible misclassification of a proportion of non-RA subjects.

In addition to body weight, many food components and beverages have been investigated in relation to RA risk, with inconsistent results [64,65]. Larger evidence exists for omega-3 fatty acids, which have been associated with lower risk of developing ACPA [66] and lower odds of developing inflammatory arthritis in ACPA-positive healthy subjects [67]. Despite such promising data, however, individual food and nutrients are unlikely to confer strong effects compared with the overall diet. Accordingly, in the NHS and NHSII cohorts, despite the lack of significant relationships between intake of omega-3 fatty acids and incident RA, healthy diet calculated according to the Alternative Healthy Eating Index (AHEI) score was shown to be protective for autoantibody-positive RA in women ≤55 years of age [68]. Among the individual components of the AHEI score, lower red meat intake and moderate alcohol consumption were found to be mostly associated with decreased early onset RA risk. Interestingly, the protective dose-dependent effect of alcohol on risk for RA has been confirmed by several studies [64,65] and is similar for ACPA-positive and -negative RA [69].

### 3.4. Hormonal Factors

Adult women experience three phases of endogenous hormonal shifts in life: pregnancy, postpartum, and menopause. It has been observed that the incidence of RA is lower during pregnancy [70,71], which might be explained by the complex mechanisms of immune tolerance and activation of immunoregulatory pathways [72]. Interestingly, although RA overall ameliorates in the course of pregnancy [73], pregnancy-related improvements of the disease are more evident in ACPA-negative RA [74]. Three to 24 months after delivery, the risk of RA seems to be increased [70,75], possibly due to the drastic fall in hormonal levels and increased prolactin during breastfeeding. Although detailed stratification based on the autoantibody serotype is not available, one study has suggested that the increased postpartum risk might be restricted to ACPA-negative RA [76]. The relation between parity and risk of incident RA is more difficult to establish, with recent meta-analyses providing conflicting results [77,78]. Previous pregnancy and childbirth appear overall irrelevant [78] or only slightly protective, with a J-shaped association between the number of pregnancies and RA risk [77]. However, the effect of parity might vary according to the patient’s HLA genotype. In particular, it has been reported that the protective effect of previous pregnancies is in fact restricted to RA women who possess the shared epitope [79]. Although these data need to be confirmed, it would thus appear that the positive impact of parity, if any, is more related to autoantibody-positive RA.

The third hormonal shift is during menopause, when estrogen levels decrease. In the large NHS and NHSII prospective cohorts, menopausal factors were only marginally associated with the risk of autoantibody-positive RA, whilst postmenopausal women had more than a doubled risk of autoantibody-negative disease compared with premenopausal women [80]. These results are in agreement with another smaller case-control study showing an association between early menopause and increased risk of RF-negative RA [81]. However, in the NHS and NHSII studies, the peak risk of developing autoantibody-negative RA was mostly observed after the menopausal transition [80], suggesting the contribution of additional yet unidentified factors acting together with hormonal shifts. Accordingly, the association between menopause and different RA serotypes remains controversial, with some studies rather reporting an increased risk of RF-positive RA in association with early age at menopause [82]. Ovarian failure has indeed been suggested to impact on RA-specific autoimmunity. In a female population of first-degree relatives of patients with RA, ACPA positivity was associated with menopause [83]; mechanistically, a decrease in estrogens may create a proinflammatory state characterized by low antibody sialylation as a consequence of downregulation of the enzyme β-galactoside α2,6-sialyltransferases 1 in plasmablasts [84].

### 3.5. Mental Health, Post-Traumatic Stress Disorders, and Depression

In longitudinal studies, depression has been associated with future risk of several chronic inflammatory diseases, including psoriasis and psoriatic arthritis [85,86] and inflammatory bowel diseases [87]. Two retrospective cohort studies suggested that depression may increase the risk for developing RA [88,89]. However, conclusions were limited due to possible confounding from lifestyle factors as well as possible reverse causation bias where early RA symptoms may have worsened mood prior to clinical arthritis. A recent and large nationwide longitudinal study of nearly 200,000 women with up to 22 years of follow-up found that indicators of depression (measured as a composite of self-reported clinician-diagnosed depression, regular antidepressant use, or a 5-question Mental Health Inventory score of <60) were associated with increased risk of developing incident RA [90]. Of note, the composite measure of depression was specifically associated with 63% increased risk for autoantibody-negative RA irrespective of measured factors including smoking pack-years, BMI, dietary intake, menopausal status, or physical activity. This study adds to the growing literature implicating a possible connection between RA risk and mental health in general. In the NHSII, an increasing number of symptoms of post-traumatic stress disorder (PTSD) increased RA risk [91]. Similarly, among military members, PTSD was associated with a 58% increased risk for any incident autoimmune disease, the most common being RA [92]. A significant association between PTSD and increased risk for incident RA has also been found among individuals exposed to the terrorist attack on the World Trade Center [93]. Mechanistically, patients with depression as well as individuals with post-traumatic stress disorder have increased levels of systemic inflammatory markers, such as IL-6 and C-reactive protein (CRP), compared to healthy controls, which could trigger inflammatory arthritis in predisposed subjects [94]. The reasons why such proinflammatory milieu may specifically favor the development of autoantibody-negative RA remain, however, unexplained.

### 3.6. Drugs

Studies focusing on immune-related adverse events (irAEs) of patients with cancer treated with immune checkpoint inhibitors (ICIs) may provide a good opportunity to unravel the characteristics and underlying immunological mechanisms of early stage autoantibody-negative RA. Immune checkpoint inhibitors target co-inhibitory pathways that normally function to downregulate T cell activation, such as cytotoxic T-lymphocyte antigen-4 CTLA-4, programmed death 1 (PD-1), or its ligand PD-L1. By blocking these co-inhibitory pathways, ICIs promote T cell-mediated antitumor immunity but may lead to a break in self-tolerance [95], manifesting as systemic or organ-specific autoimmunity. Inflammatory arthritis (IA) is the most common rheumatic irAE and has the potential to persist even after ICI cessation. IA-irAE is characteristically polyarticular and autoantibody negative [96]. A retrospective, cross-sectional comparative study on 20 cancer patients with de novo IA of the peripheral joints after the initiation of ICI therapy (ipilimumab, nivolumab, pembrolizumab, or combination) found that IA-irAE resembled autoantibody-negative RA in certain immunological characteristics, including negativity for RF and ACPA and a speckled pattern of anti-nuclear antibodies [97]. The low prevalence or very low titers of RF and ACPA in patients with IA-irAE has also been reported by others [98,99,100]. Based on these findings, one could hypothesize that IA-irAE is a disease process more likely dependent on autoreactive T cells rather than on B cell-based autoantibody production, and that dysregulation of PD-1 signaling may play an important role in autoantibody-negative RA.

## 4. Immunopathogenesis

### 4.1. Autoantibodies

The progressive improvement in autoantibody laboratory assays, together with the recognition of other possible autoantibody specificities beyond RF and ACPA, have led some authors to hypothesize that at least a proportion of autoantibody-negative RA is in fact incorrectly denominated seronegative [9]. Apart from non-canonical ACPAs, which can be detected in as many as 16% of ACPA-negative patients by multiplex citrullinated peptide arrays [101], the occurrence of autoantibody responses to other post-translational modified proteins is a well-known phenomenon in RA (Table 1). This is the case, for instance, of autoantibodies against carbamylated proteins (anti-CarP), reported to be present in 10–15% of RF and ACPA double-negative patients [102,103]. In contrast with the well-established clinical and pathogenetic role of ACPA [104,105], the significance of anti-CarP in the context of ACPA negativity remains controversial [106,107,108]. Still, the strong and specific association of HLA-B*08 carrying aspartate at position 9 with anti-CarP but not ACPA [109] reinforces the concept that the spectrum of autoimmunity in RA is broader than that traditionally recognized. The discovery of additional reactivities outside the spectrum of anti-modified protein autoantibodies, such as those against peptidylarginine deiminases (PADs), adds a further layer of complexity to the understanding of the immunological nature of autoantibody-negative RA. Both anti-PAD4 and anti-PAD3 may indeed be found in a variable proportion of 3–19% of ACPA-negative patients, and appear associated with worst disease prognosis in terms of joint damage and extra-articular involvement [110]. More sophisticated techniques, such as high-density protein microarrays, have very recently identified a number of other possible autoantibody specifities in approximatively 35% of ACPA-negative patients, with specificity of >90% for RA [111]. Of these, the combination of anti-PTX3 and anti-DUSP11 was found to have optimal diagnostic performance regardless of ACPA status. Whilst the exact role of DUSP11 is poorly studied, PTX3 is an essential component of innate immunity [112], underscoring the possible differential contribution of different branches of the immune system in autoantibody-negative compared to -positive RA.

### 4.2. Cytokine Networks

Cytokine-mediated pathways are at the center of the immunopathogenesis of RA, as demonstrated by animal models of arthritis and, more importantly, as confirmed by the dramatic improvement of patients’ prognosis upon the introduction of anti-cytokine therapies [113]. The pleiotropy, redundancy, synergy, and antagonism of the cytokine system hamper the establishment of linear models of cytokine hierarchy across multiple stages or phenotypes of the disease. However, the partially different response to therapies among individual patients with the same formal diagnosis of RA indicates that a molecular taxonomy based on cytokine ‘hubs’ could better address possible pathophysiological differences within the disease [114]. The efficacy of tumor necrosis factor (TNF)-α inhibition irrespective of the autoantibody status [115] indicates that this cytokine likely represents a common effector pathway that acts downstream of many inflammatory processes. Still, the selective benefit of concomitant methotrexate only in patients who are autoantibody positive [116] raises the possibility that the sources and effector functions of TNF-α may differ between the two disease subtypes, with autoantibody-negative RA more dependent on myeloid production, and less in need of simultaneous inhibition of IL-6-mediated B cell activation [117,118]. In line with this concept, myeloid-driven synovial and systemic inflammation appear more susceptible to TNF-α rather than IL-6R antagonism [119], and even less responsive to B cell-depleting agents [120]. Equally important, in a proportion of patients with abrupt onset polyarticular synovitis and systemic symptoms, as often occurs in autoantibody-negative RA, the pathogenetic events might be more centered on activation of the inflammasome and hyperproduction of IL-1β [8,121]. Accordingly, similarly to systemic adult-onset Still’s disease, these patients may exhibit a response to IL-1 blockers [122], which in contrast have limited efficacy in classical autoimmune RA. Further complicating the puzzle of cytokine taxonomy in RA, response has also been noted with IL-17A inhibition [123], suggesting that some forms of (autoantibody-negative) RA may share similarities with psoriatic arthritis [8].

### 4.3. Synovial Pathology

Irrespective of the clinical diagnosis, the synovial membrane in the course of chronic inflammatory arthritis is characterized by a number of common histopathological changes, including neoangiogenesis, proliferation and activation of the tissue stroma, and infiltration of the sublining layer by cells of the innate and adaptive immune system [124,125,126,127]. Notwithstanding these general similarities, synovial inflammatory features greatly vary in terms of cellular composition and reciprocal spatial arrangements across different disease entities and within a same disease, possibly reflecting divergent pathogenetic pathways [128,129]. One of the best characterized immunopathological and molecular feature is the relative balance between infiltrating macrophages and lymphoid cells, which helps to distinguish among myeloid, lympho-myeloid, and pauci-immune synovial pathotypes [130]. Relevantly, the lympho-myeloid pathotype is enriched in B cells and in genes of the adaptive immune response both locally [130,131] and systemically [119,132,133], and associates with a poorer response to non-targeted treatments [119,120,132,134] and more severe radiographic damage [130,131,133,134,135,136].

Despite B cell lineage synovial and systemic signatures predominating in patients with positive autoantibodies, particularly ACPA [130,131,132,133,135], autoantibody-negative RA can still display features indicative of adaptive immune responses at the site of inflammation in a variable but significant proportion of the cases [130,131,133,137] (Table 1) and, at least in long-standing disease, B cell-rich synovitis can be detected in as many as 45% of patients, without significant differences compared with autoantibody-positive RA [135]. Importantly, this finding has also been confirmed at the single-cell level, where different B cell subpopulations including memory and plasma cells appear mostly comparable irrespective of the autoantibody status [138,139]. It is therefore possible that, although the initial pathogenetic pathways of autoantibody-positive and -negative RA may differ, the two disease subsets eventually convey on shared inflammatory mechanisms. Confirming this notion, psoriatic arthritis, which is traditionally recognized as a non-autoimmune condition, may show some aspects of B cell activation in the synovium [140], and does not significantly differ from autoantibody-negative RA in the extent of B cell infiltration [141]. Single-cell sequencing of synovial immune cells has, however, very recently revealed key differences in local gene expression, with lower antigen processing and presentation activity and lower cytotoxicity and exhaustion in B and T lymphocytes but increased pro-inflammatory activity in macrophages from ACPA-negative compared to ACPA-positive RA [139] (Table 1). If confirmed, such pathogenetic diversity could help to explain the different response to targeted drugs with various mechanisms of action [104] and fuel precision therapy based on the autoantibody status.

### 4.4. Extra-Articular Involvement

Patients with RA suffer from multiple comorbidities, including cardiovascular disease (CVD), interstitial lung disease, and accelerated systemic bone loss [142]. Although chronic inflammation represents the major pathogenetic process of RA extra-articular involvements [143], the higher comorbid burden recognized in autoantibody-positive patients since the very beginning of the disease [144,145,146] and also beyond inflammation [2,147,148] underlines the importance of additional factors, including adaptive immune mechanisms and autoantibodies. However, at least in the case of CVD, incident cardiovascular events may occur early during the course of the disease also in autoantibody-negative RA [149]. Whether such an association generally arises from traditional risk factors or might be more specific of proinflammatory cytokines and inflammasome-driven pathways [150] remains to be established. Of particular relevance to autoantibody-negative RA, depression not only increases the risk of disease development, but also complicates its course in a significant proportion of the patients [151]. From a pathophysiologic perspective, TNF-driven arthritis, which is a model of myeloid cell-mediated disease, is associated with activation of microglia and neuroinflammation of certain brain regions, a pattern that is not seen in lymphoid cell-based arthritis [152]. A better understanding of the pathogenetic pathways underlying the development of different comorbidities in different subtypes of RA will certainly translate into more personalized medical approaches.

## 5. Conclusions

**Figure 1:**
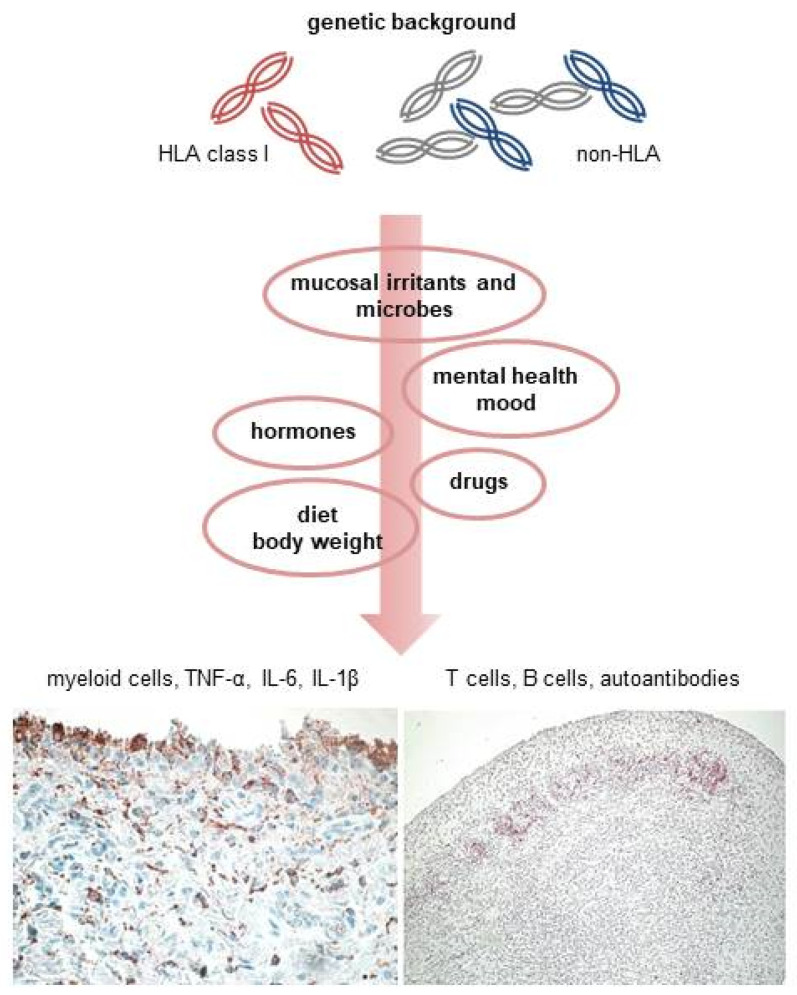
Proposed model of disease pathogenesis in autoantibody-negative rheumatoid arthritis.

The mechanisms lying behind the phenotypic heterogeneity of RA remain largely unidentified, but possible genetic, environmental, and pathogenetic differences in relation to the autoantibody status are starting to emerge (Table 1). The timeline of disease development is remarkably less characterized in autoantibody-negative RA, but genetic susceptibility (mostly outside the HLA region) and environmental and endogenous triggers contribute to the development of an immune-inflammatory cascade that appears, at least in a proportion of the patients, largely driven by innate immune pathways and myeloid cytokines (Figure 1). A better definition of the pathogenic taxonomy of autoantibody-negative RA is needed to help improve the clinical management of this underlooked disease subtype, which remains challenging even in the modern treatment era.

The genetic susceptibility risk of autoantibody-negative rheumatoid arthritis mostly relies on class I HLA genes and a number of yet unidentified non-HLA loci. On the background of variable genetic predisposition, multiple exogeneous, endogenous, and stochastic factors contribute to the onset of the inflammatory cascade. These include, but are not limited to, inflammatory and microbial stimuli at mucosal sites, dietary factors and excess body weight, hormonal factors, mental health, and medications. In a proportion of the patients, the immunopathology of synovitis, at least in the initial stages, appears largely myeloid driven, with an abundant production of proinflammatory cytokines and only minor involvement of cells of the adaptive immune system. However, T and B cells can infiltrate the synovial tissue, and autoantibody production may also occur.

## Figures and Tables

**Table 1:** Genetic, environmental, and immunopathologic differences between autoantibody-positive and -negative rheumatoid arthritis.

	Autoantibody-Positive	Autoantibody-Negative
Genetic associations	shared epitope-containing HLA-DRB1 alleles	HLA-B*08 with aspartate at position 9 and DRB1*03 with serine at position 11 non-HLA genes
Environmental and endogenous factors	Smoking silica air pollution organic dusts asbestos pulmonary inflammation dysbiosis early menopause	air pollution organic dusts asbestos pulmonary inflammation dysbiosis excess body weight postpartum early menopause depression post-traumatic stress immune checkpoint inhibitors
Autoantibodies Anti-CarP Anti-acetylated Anti-PAD	15–65% 40–60% 2–18%	10–15% 10–25% 3–19%
Cytokines	TNF-α (lymphoid) IL-6	TNF-α (myeloid) IL-6 IL-1β
Synovial pathology	mostly lympho-myeloid pattern higher levels of CD19+ B cells, CD3+ T cells, lymphoid aggregates, germinal centers	lympho-myeloid, diffuse-myeloid and pauci-immune pattern lower antigen processing and presentation activity in B cells lower cytotoxic and exhausted gene expression in T cells higher proinflammatory cytokine expression in macrophages

## Data Availability

Not applicable.
